# Cux2 refines the forelimb field by controlling expression of *Raldh2* and *Hox* genes

**DOI:** 10.1242/bio.040584

**Published:** 2019-01-16

**Authors:** Shogo Ueda, Ingrid Rosenburg Cordeiro, Yuuta Moriyama, Chika Nishimori, Kei-ichi Kai, Reiko Yu, Ryoichiro Nakato, Katsuhiko Shirahige, Mikiko Tanaka

**Affiliations:** 1 Department of Life Science and Technology, Tokyo Institute of Technology, Midori-ku, Yokohama, 226-8501, Japan; 2 Research Center for Epigenetic Disease, Institute of Molecular and Cellular Biosciences, University of Tokyo, Bunkyo-ku, Tokyo, 113-0032, Japan

**Keywords:** Forelimb field specification, Forelimb bud initiation, Cux2

## Abstract

In vertebrates, two pairs of buds that give rise to the fore- and hindlimbs form at discrete positions along the rostral-caudal axis of the body. The mechanism responsible for the positioning of the limb buds is still largely unknown. Here we show a novel function for Cut homeobox transcription factor 2 (Cux2), the ortholog of *Drosophila* cut, in refining the forelimb field during chick development. *Cux2* is expressed in the forelimb field before the emergence of the limb buds. Knocking down the expression of *Cux2* using small interfering RNA (siRNA) resulted in a caudal shift of the forelimb bud, whereas misexpression of *Cux2* or the constitutively active *Cux2*-*VP16* caused a rostral shift of the forelimb bud or reduction of the forelimb field along the anterior-posterior axis. Further functional analyses revealed that expression of *Hoxb* genes and *retinaldehyde dehydrogenase 2* (*Raldh2*), which are involved in limb positioning, are directly activated by Cux2 in the lateral plate mesoderm. Our data suggest that Cux2 in the lateral plate mesoderm refines the forelimb field via regulation of *Raldh2* and *Hoxb* genes in chicken embryos.

## Abstract (precis)

**Summary:** Cux2 in the lateral plate mesoderm refines the forelimb field via regulation of *Raldh2* and *Hoxb* genes in chicken embryos.

## INTRODUCTION

Limb buds emerge as small bulges that protrude from the body trunk at discrete positions along the rostral-caudal axis. It has been proposed that the nested expression of *Hox* genes in the lateral plate mesoderm is related to the specification of position along the rostral-caudal axis to generate forelimb, interlimb and hindlimb regions (Burke, 2000; Burke et al., 1995; Cohn et al., 1995, 1997). Such a role of *Hox* genes in limb positioning is supported by mice lacking *Hoxb5*, which show a rostral shift of the forelimb buds (Rancourt et al., 1995). Recent studies provided evidence for a role for *Hox* genes in the specification of the forelimb field via regulation of the transcription of *T-box 5* (*Tbx5*). *Tbx5* is expressed in the anterior paired appendages of zebrafish, chick and mouse embryos (Gibson-Brown et al., 1996; Isaac et al., 1998; Tamura et al., 1999) and plays dispensable roles in the initiation of limb development (Ahn et al., 2002; Garrity et al., 2002; Naiche and Papaioannou, 2003; Ng et al., 2002; Rallis et al., 2003; Takeuchi et al., 2003). Developmental and molecular analyses of chick and mouse embryos showed that, in the forelimb field, rostrally expressed *Hox* genes directly activate *Tbx5* transcription and thus control the position of the forelimb field (Minguillon et al., 2012). Furthermore, caudally expressed *Hoxc9*, which is expressed in the interlimb and hindlimb fields, represses expression of *Tbx5* in the caudal lateral plate mesoderm, possibly by recruiting co-repressors (Nishimoto et al., 2014). Rostrally expanded distribution of Hoxc8 is present in the body trunk of the python snake (Cohn and Tickle, 1999), supporting the view that Hoxc8 represses expression of *Tbx5* (Nishimoto et al., 2014). In fact, the position of the hindlimbs shift posteriorly in *Hoxc8* null mutants (van den Akker et al., 2001). These results suggest that a combination of collinearly expressed *Hox* genes dictates the position of forelimbs along the rostral-caudal axis (Nishimoto et al., 2014).

Recent analyses of mouse mutants revealed that *Hox9* and *Hox5* genes are involved in establishing the posterior and anterior field of the forelimb, respectively (Xu et al., 2013; Xu and Wellik, 2011). The early polarity in the limb field is established by antagonistic interaction between *Hand2* in the posterior mesenchyme and *Gli3* in the anterior mesenchyme (Welscher et al., 2002b), prior to the initiation of *Shh* expression, which marks the zone of polarizing activity in the posterior margin of the limb buds (Riddle et al., 1993). An analysis of *Hox9* quadruple mutants revealed that axial *Hox9* paralogs are involved in the establishment of the posterior forelimb field by triggering the posteriorly restricted expression of *Hand2*, which regulates *Shh* directly to initiate its expression in the posterior margin of the limb bud (Xu and Wellik, 2011). In contrast, deletion of all three *Hox5* genes suggests that Hox5 proteins interact with promyelocytic leukemia zinc finger (Plzf) and cooperatively mediate repression of *Shh* expression in the anterior part of the forelimb buds (Xu et al., 2013).

Past and recent studies indicated the involvement of retinoic acid in the initiation and specification of the forelimb field. Administration of disulfiram, an inhibitor of retinoic acid synthesis, to chick embryos prior to limb bud outgrowth leads to hypoplasia or a caudal shift of the forelimb bud (Stratford et al., 1996). In mouse *retinaldehyde dehydrogenase 2* (*Raldh2*) mutants, the heart-forming field is expanded posteriorly and forelimb initiation fails (Ryckebusch et al., 2008; Sirbu et al., 2008; Zhao et al., 2009). Similarly, zebrafish mutants for *raldh2* fail to initiate pectoral fin formation (Begemann et al., 2001), and zebrafish embryos treated with the retinoic acid inhibitor 4-diethylaminobenzaldehyde (DEAB) show a posterior expansion of the heart field and lack pectoral fin buds (Waxman et al., 2008). Several lines of evidence indicate that retinoic acid signaling regulates the transcription of *Hox* genes and leads to the regionalization of the lateral plate mesoderm along the anterior-posterior axis (Lo and Frasch, 2003; Niederreither et al., 1999; Waxman et al., 2008; Xavier-Neto et al., 2000). More recently, developmental analyses of chick and mouse embryos revealed that retinoic acid signaling and Hox proteins cooperatively activate *Tbx5* transcription to induce forelimb bud formation (Nishimoto et al., 2015).

Cut/Cux transcription factors have four conserved DNA binding domains, three cut repeats and a homeodomain (Gingrasa et al., 2005; Hulea and Nepveu, 2012; Sansregret and Nepveu, 2008). In *Drosophila*, *cut* is expressed in the dorso-ventral boundary cells of the forelimb disc (Blochlinger et al., 1993; Buceta et al., 2007; Micchelli et al., 1997), and depletion of cut function disrupts the formation of the forelimb margin, suggesting that cut is required for dorso-ventral boundary formation of the developing forelimb margin (Blochlinger et al., 1993; Buceta et al., 2007; Micchelli et al., 1997). In mouse and chicken, two orthologs of cut, Cux1 and Cux2, have been identified (Tavares et al., 2000; Valarche et al., 1993). In chick embryos, *Cux1* is expressed in the ectoderm adjacent to the apical ectodermal ridge and restricts its position within the limb buds (Tavares et al., 2000). While *Cux2* is initially expressed in the presumptive forelimb field, its expression becomes restricted to the posterior part of the limb buds and to the interlimb flank of chick embryos (Tavares et al., 2000). The function of Cux2 in the lateral plate mesoderm, however, remains to be identified.

Here we explored the function of Cux2 during chick embryogenesis. We show that Cux2 is involved in the specification of the forelimb field. Further functional analyses revealed that Cux2 directly activates the expression of *Raldh2* and *Hoxb* genes, which are involved in the specification of the limb-forming fields. These results suggest that Cux2 in the lateral plate mesoderm refines the forelimb-forming fields via regulation of transcription of *Raldh2* and *Hoxb* genes in chicken embryos.

## RESULTS

### Cux2 is involved in specification of forelimb fields

First, we investigated the expression of *Cux2* during development of chick embryos (Fig. S1A–E). Transcripts of *Cux2* were detected throughout the lateral plate mesoderm at Hamburger-Hamilton stage (HH) 13 (Fig. S1A), and subsequently expression was increased in the forelimb-forming fields (at the level of somites 14–18) at early HH 14 (20-somite stage; Fig. S1B). Expression of *Cux2* in the anterior part of the forelimb field gradually decreased, and *Cux2* expression became restricted to the posterior part of the forelimb buds and interlimb flank region by HH 17; weak expression was also detected in the posterior part of the hindlimb buds at the same stage (Fig. S1C). *Cux2* was expressed in the posterior limb buds and interlimb flank region by HH 19 (Fig. S1D). By HH 26, transcripts of *Cux2* were undetectable in the interlimb flank and were restricted to the posterior limb buds (Fig. S1E).

Expression analyses of *Cux2* showed that it is expressed in the limb-forming region prior to the outgrowth of limb bud. To investigate whether *Cux2* has a role in limb development, we downregulated endogenous *Cux2* using siRNAs that targeted chick *Cux2* (*Cux2*-siRNA). To evaluate the effect of *Cux2*-siRNA *in vitro*, we transfected COS7 cells with *Cux2*-siRNA or control-siRNA together with pCMV-*Cux2*-*EGFP* (Fig S1F–H). At 24 h after transfection with *Cux2*-siRNA, the number of EGFP-positive cells was reduced by 40% relative to the control siRNA (*P*<0.00001, Student's *t*-test; Fig. S1H).

We then investigated the effect of depleting *Cux2* in limb development (Fig. S1I,J). At 22–24 h after electroporation of *Cux2*-siRNA into the presumptive forelimb region of HH 15–17 chick embryos, expression of *Cux2* was relatively downregulated in the EGFP-positive region (2/6; Fig. S1I). In contrast, *Cux2* expression was not affected in the forelimb buds of embryos electroporated with control-siRNA (0/9; Fig. S1J).

**Fig. 1.**
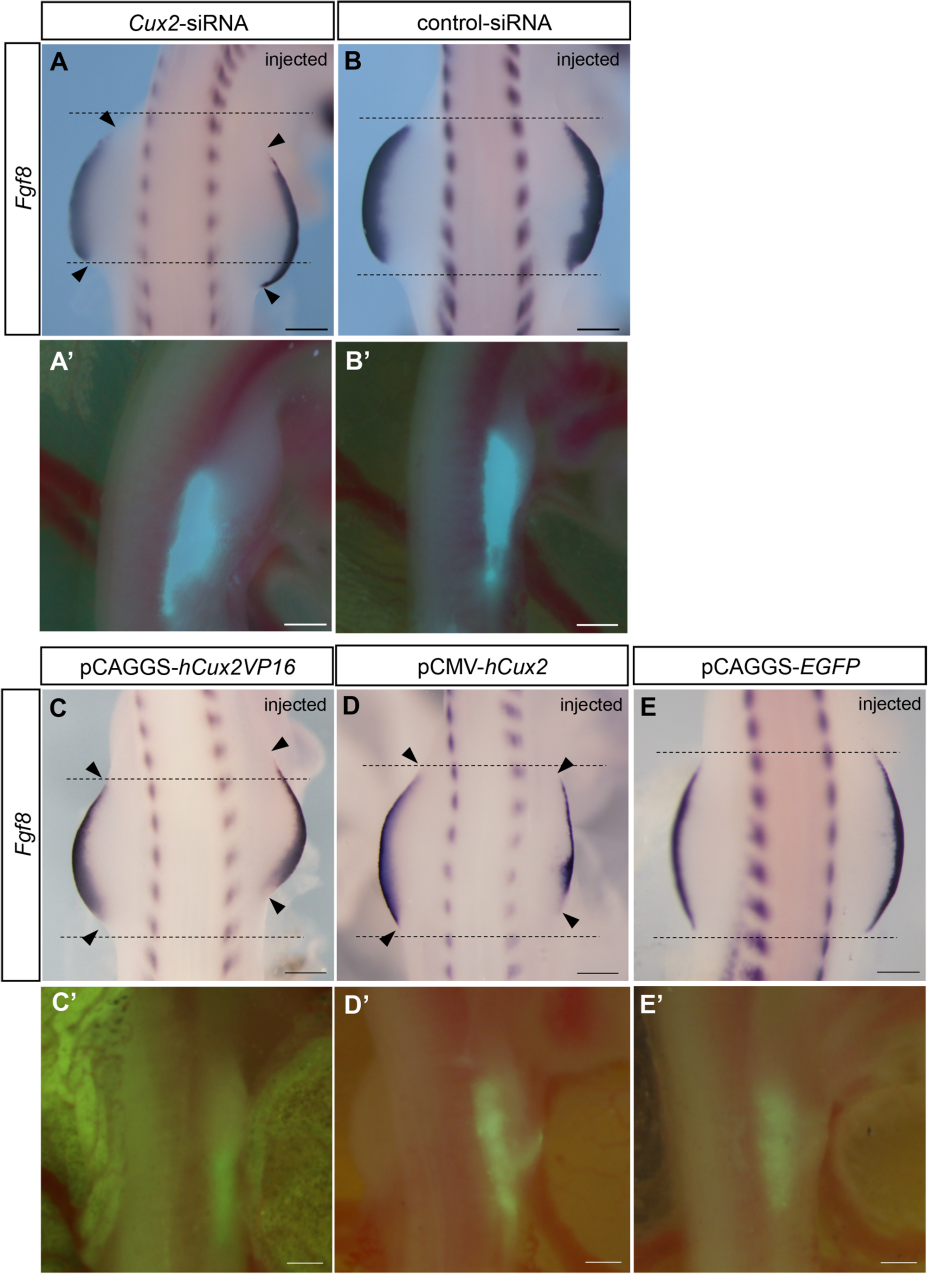
**Cux2 is involved in refining the forelimb field in chick embryos.** (A,B) Expression of *Fgf8* in forelimb buds after electroporation of *Cux2*-siRNA (A) or control-siRNA (B). The expression of *Fgf8* was extended posteriorly on the electroporated right side (A), whereas that of control embryos was unchanged (B). (C) Expression of *Fgf8* in forelimb buds after electroporation of pCAGGS-*hCux2-VP16*. *Fgf8* expression is shifted anteriorly. (D) Expression of *Fgf8* in forelimb buds after electroporation of pCMV-*hCux2*. *Fgf8* expression is reduced along the anterior-posterior axis. (E) Expression of *Fgf8* in forelimb buds after electroporation of pCAGGS-*EGFP*. Control embryos did not show altered *Fgf8* expression (E). Black dotted lines indicate the width of the left (control side) forelimb bud. Arrowheads indicate the anterior and posterior end of the forelimb bud. (A′–E′) pCAGGS-*EGFP* was used to assay efficiency of electroporation. Scale bars: 500 μm.

To investigate the role of *Cux2* in forelimb development, we then co-electroporated *Cux2*-siRNA or control siRNA with pCAGGS-*EGFP* into the presumptive forelimb field on the right side of HH 15–17 embryos (Fig. 1A,B). The embryos were fixed 22–24 h after electroporation and examined for expression of *Fgf8*, a marker for the apical ectodermal ridge of limb buds (Crossley et al., 1996). When *Cux2*-siRNA was introduced into the presumptive forelimb field, 8 of 19 embryos showed changes in the posterior extent of the electroporated forelimb bud (Fig. 1A; Fig. S2). Embryos in which the control-siRNA was electroporated did not show any morphological changes in forelimb formation (0/9; Fig. 1B; Fig. S2). These results suggest that Cux2 is involved in the development of the forelimb buds.

We then electroporated constitutively active human *Cux2* (*hCux2*)-*VP16* or full-length *hCux2* constructs into the presumptive forelimb field at HH 13–14 to further investigate the function of Cux2 in limb development. The morphology of the limb buds was evaluated based on the expression of *Fgf8* 22–24 h after electroporation (Fig. 1C–E). Misexpression of *hCux2*-*VP16* led to an anterior shift of the forelimb buds, or a reduction in the forelimb field along the anterior-posterior axis (7/14; Fig. 1C; Fig. S2). Similarly, electroporation of full-length *hCux2* led to an anterior shift of the posterior boundary of the forelimb buds (4/10; Fig. 1D). We also found one embryo with posteriorly extended forelimb bud, after electroporation of full-length *hCux2.* Control forelimb buds electroporated with pCAGGS-*EGFP* did not show altered limb bud morphology (0/10; Fig. 1E; Fig. S2). These results indicate that Cux2 is involved in specification of the forelimb field.

Despite the anterior shift of forelimb buds at an early stage, we could not detect any gross changes in the cartilage patterns for *hCux2-VP1*6-misexpressing forelimbs 8 days after electroporation (*n*=11; Fig. S3). Thus, the effects of *Cux2* misexpression are transient, and the forelimb buds normalize.

### Misexpression of *Cux2* Alters *Hand2* and *Shh* expression

**Fig. 2.**
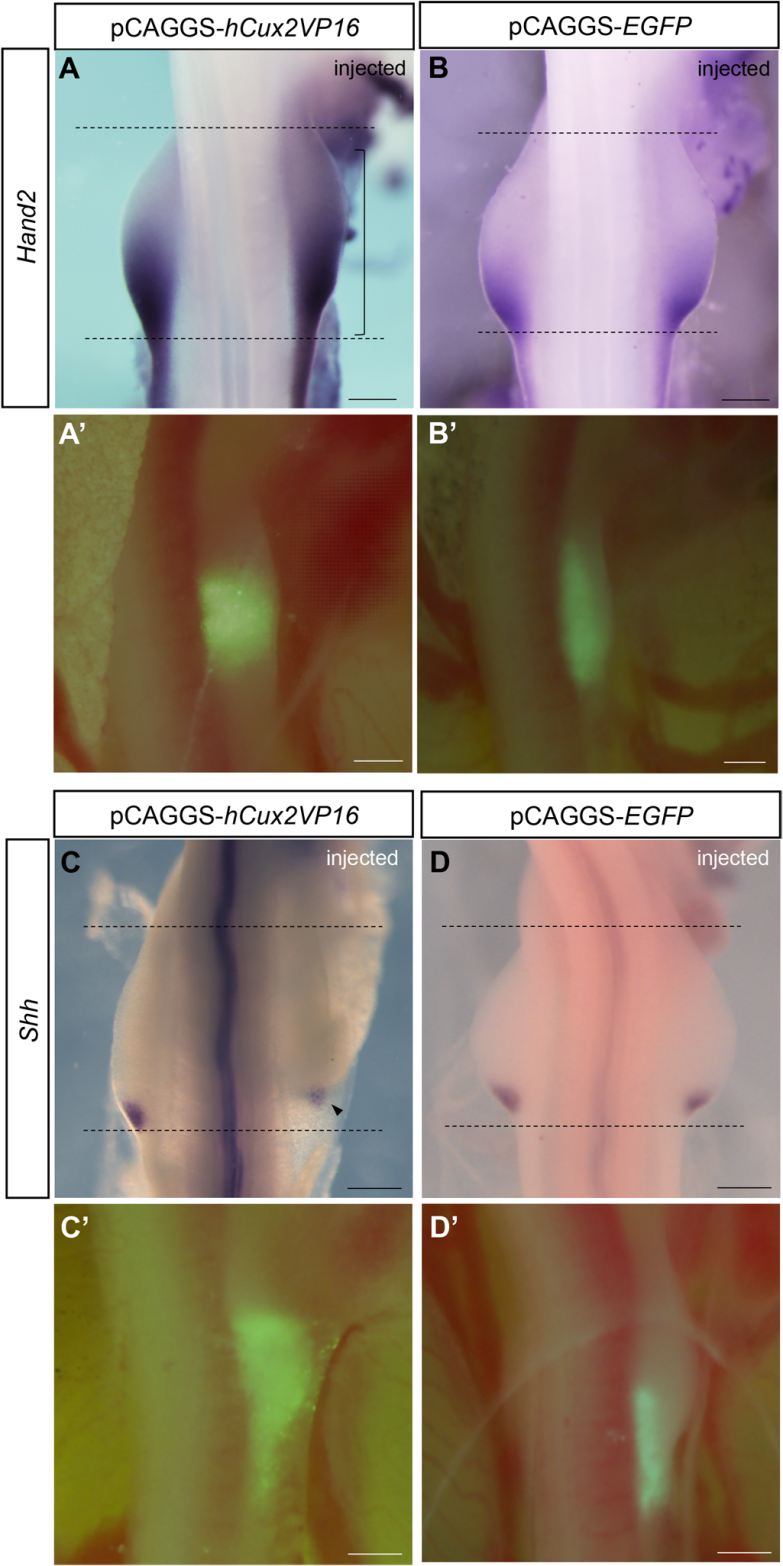
**Cux2 alters expression of *Hand2* and *Shh*.** (A–D) Expression of *Hand2* (A,B) and *Shh* (C,D) in forelimb buds after electroporation of pCAGGS-*hCux2-VP16* (A,C) or pCAGGS-*EGFP* (B,D). Misexpression of *hCux2-VP16* led to the expansion of *Hand2* expression throughout the limb bud (a bracket in A) and the rostral shift in *Shh* expression (an arrowhead in C), but electroporation of control *EGFP* constructs did not alter expression of *Hand2* (B) or *Shh* (D). Black dotted lines indicate the width of the left (control side) forelimb bud. (A′–D′) pCAGGS-*EGFP* was used to assay efficiency of electroporation. Scale bars: 500 μm.

Signals involved in pre-patterning along the anterior-posterior axis of the limb and establishing the polarizing region have been proposed to also be involved in limb positioning along the rostral-caudal body axis (Rallis et al., 2003). To investigate whether *Cux2* is involved in the anterior-posterior patterning of the limb, we examined the expression of *Hand2* and *Shh* after *Cux2* misexpression (Fig. 2). *hCux2*-*VP16* was mixed with pCAGGS-*EGFP,* and electroporated into the right side of the forelimb field at HH 13-14, and embryos were fixed at 22–24 h after electroporation for gene expression analysis. Misexpression of *hCux2*-*VP16* led to the anterior expansion of *Hand2* throughout the forelimb field on the injected side (3/9) only in embryos with shifted limbs (three out of three shifted limbs), whereas *Hand2* expression was restricted to the posterior domain on the uninjected side (3/9; Fig. 2A). No changes in *Hand2* expression were observed after misexpression of the control pCAGGS-*EGFP* construct alone (0/5; Fig. 2B). Additionally, misexpression of *hCux2*-*VP16* led to a rostral shift in the expression of *Shh* (3/6) only in embryos with shifted limbs (two out of three shifted limbs; Fig. 2C) and, of these, two samples showed reduced *Shh* expression (2/3; Fig. 2C), whereas misexpression of the control pCAGGS-*EGFP* construct did not alter *Shh* expression (0/6; Fig. 2D). These results suggest that Cux2 may be involved in establishment of the position of *Shh* expression in the polarizing region, at least in part via regulation of *Hand2* expression.

### Cux2 directly activates the expression of *Raldh2* and *Hoxb* in the lateral plate mesoderm

**Fig. 3.**
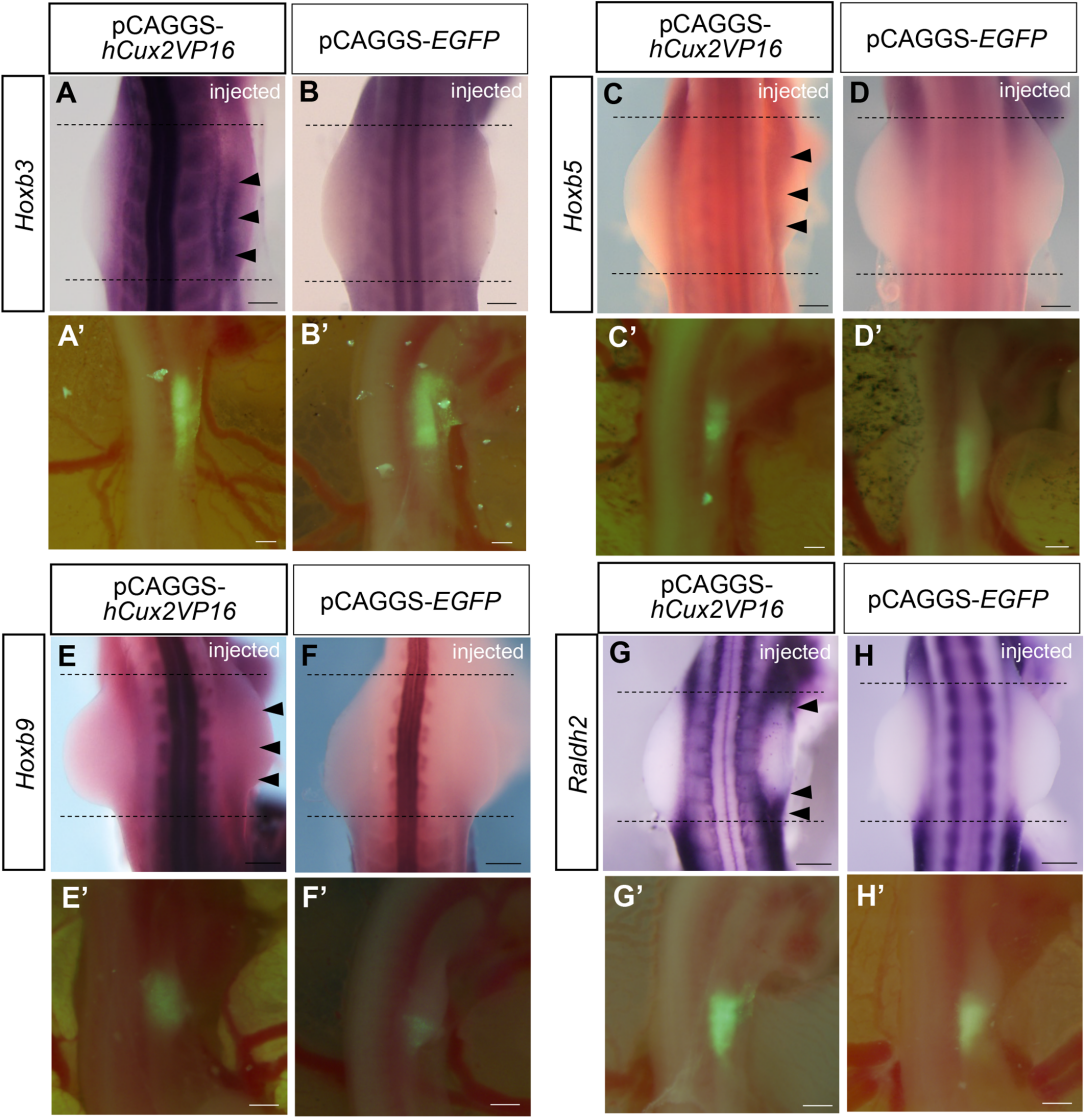
**Cux2 regulates expression of *Hoxb* genes and *Raldh2* in forelimb buds.** (A–H) Expression of *Hoxb3* (A,B), *Hoxb5* (C,D), *Hoxb9* (E,F), and *Raldh2* (G,H) in forelimb buds after electroporation of pCAGGS-*hCux2-VP16* (A,C,E,G) or pCAGGS-*EGFP* (B,D,F,H). (A–F) *Hoxb3* was anteriorly expanded to the forelimb bud (arrowheads in A), *Hoxb5* was ectopically expressed in the forelimb bud (arrowheads in C), and weak upregulation of *Hoxb9* was seen throughout the forelimb bud (arrowheads in E) after electroporation of pCAGGS-*hCux2-VP16*, whereas *Hoxb3,b5* or *b9* expression was not altered in forelimb buds of control embryos (B,D,F). (G,H) Expression of *Raldh2* was activated in embryos electroporated with pCAGGS-*hCux2-VP16* (arrowheads in G), whereas *Raldh2* expression in control embryos did not show any changes (H). Black dotted lines indicate the width of the left (control side) forelimb bud. (A′–H′) pCAGGS-*EGFP* was used to assay efficiency of electroporation. Scale bars: 500 μm.

Our results suggest the possible function of Cux2 in positioning of forelimb field, upstream of *Hand2* expression, along the anterior-posterior axis. We then explored whether Cux2 controls factors regulating *Hand2* transcription. In mouse forelimb buds, *Hox9* is involved in the positioning of *Shh* expression via regulation of Hand2 expression (Xu and Wellik, 2011). Furthermore, *Hoxb5* is involved in forelimb positioning (Rancourt et al., 1995). Thus, we examined the possibility that Cux2 regulates expression of *Hoxb* genes (Fig. 3A–F). First, we examined whether expression of *Hoxb* genes in the lateral plate mesoderm of HH 14 and 19 chick embryos (Fig. S4), to see whether their expression overlapped with that of *Cux*2. *Hoxb3* was expressed in the anterior lateral plate mesoderm including the forelimb field at HH 14 (Fig. S4A) and in the proximal part of the limb buds as well as in the interlimb flank lateral plate mesoderm at HH 19 (Fig. S4E). *Hoxb5* transcripts were detected in the anterior lateral plate mesoderm at HH 14 (22-somite stage; Fig. S4B) and were expressed in the proximal part of the limb buds and in the interlimb flank region at HH 19 (Fig. S4F). *Hoxb9* was expressed in the posterior lateral plate mesoderm including the posterior part of forelimb field at HH 14 (Fig. S4C) and in the interlimb flank region at HH 19 (Fig. S4G). Thus, expression domains of *Hoxb3*, *Hoxb5* and *Hoxb9* are at least partially overlapping with those of *Cux2* in the lateral plate mesoderm at HH 14 and 19 (Fig. S4).

Retinoic acid is known to control expression of *Hox* genes (Conlon and Rossant, 1992; Kessel and Gruss, 1991), and the specification of forelimb field (Begemann et al., 2001; Niederreither et al., 1999; Nishimoto et al., 2015; Stratford et al., 1996; Zhao et al., 2009). We, thus, examined the possibility that Cux2 controls expression of *Raldh2*, encoding an enzyme that catalyzes the retinoic acid synthesis from retinaldehyde (Fig. 3G,H). *Raldh2* was expressed in the anterior part of the lateral plate mesoderm including the presumptive forelimb field at HH 14 (Fig. S4D) and in the interlimb flank region at HH 19 (Fig. S4H) as previously reported (Swindell et al., 1999), suggesting its expression is also overlapping with *Cux2* expression in the lateral plate mesoderm at these stages.

Next, we investigated whether *Hoxb* genes and *Raldh2* are downstream targets of Cux2 in the lateral plate mesoderm. For this purpose, we misexpressed constitutively active *Cux2* in the right side presumptive forelimb field at HH 13–14 and examined the expression of *Hoxb* genes and *Raldh2* (Fig. 3; Fig. S5). Misexpression of *Cux2*-*VP16* in the mesenchyme of the presumptive forelimb field induced anterior expansion of *Hoxb3* (3/5; Fig. 3A; Fig. S5A), whereas misexpression of pCAGGS-*EGFP* did not alter *Hoxb3* expression (0/5; Fig. 3B). In addition, we examined expression of *Hoxb5* after misexpression of constitutively active *Cux2* in the presumptive forelimb field (Fig. 3). Misexpression of *Cux2* induced an ectopic expression of *Hoxb5* in the mesenchyme of the forelimb bud (5/10; Fig. 3C; Fig. S5B), whereas introduction of control pCAGGS-*EGFP* did not alter its expression pattern (0/5; Fig. 3D). Misexpression of *Cux2* led to the weak upregulation of the *Hoxb9* expression throughout the mesenchymal region of the forelimb bud (9/16; Fig. 3E; Fig. S5C), while control pCAGGS-*EGFP* introduction did not change its expression (7/7; Fig. 3F). *Raldh2* expression was ectopically activated in the anterior and posterior parts of forelimb buds 22–24 h after electroporation of pCAGGS-*Cux2*-*VP16* (10/15; Fig. 3G), whereas introduction of pCAGGS-*EGFP* did not cause any changes in *Raldh2* expression (0/4; Fig. 3H). These results suggest that Cux2 activates expression of *Raldh2* and *Hoxb3* in the forelimb field and probably also controls other *Hoxb* genes.

**Fig. 4.**
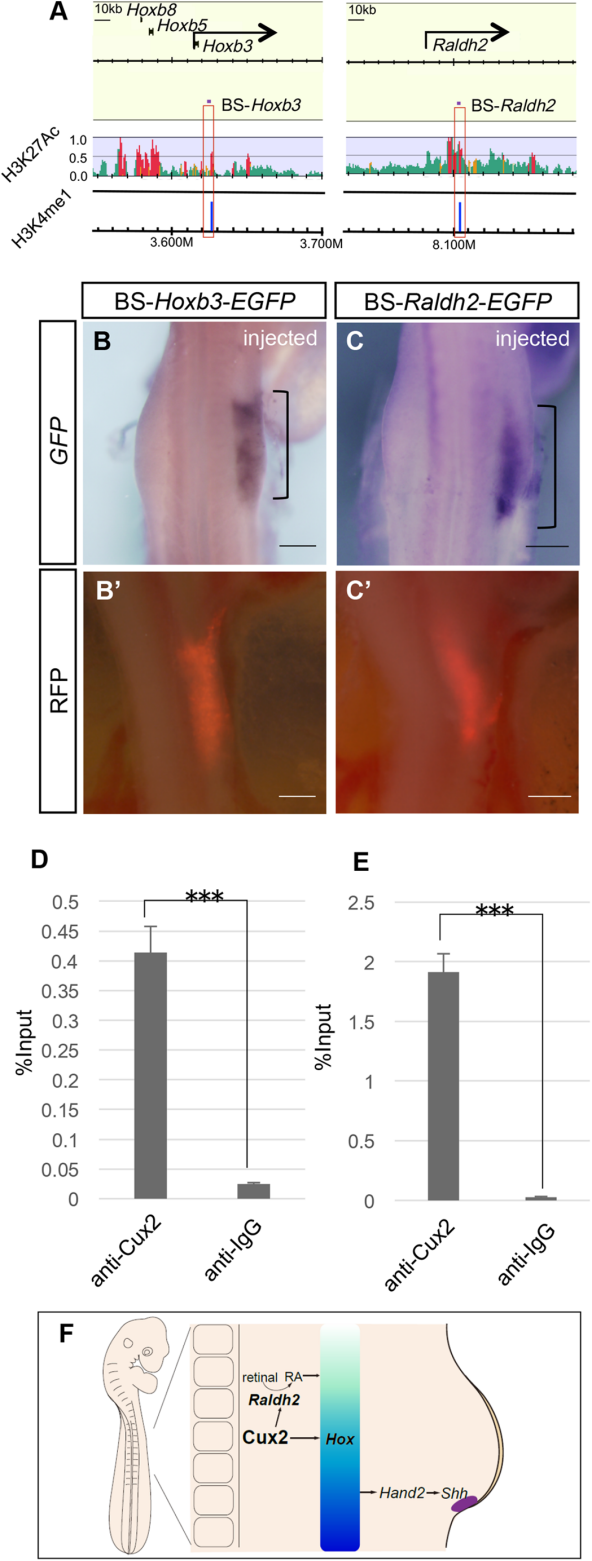
**Cux2 directly activates transcription of *Hoxb3* and *Raldh2* in the lateral plate mesoderm.** (A) ChIP-seq analysis revealed the enhancer region in the *Raldh2* and *Hoxb3* genomic landscape. H3K4me1 regions in HH-16 chicken embryo were obtained from Seki et al. (2017). Short purple bars above the red boxes indicate the 702-bp BS-*Raldh2* and 821-bp BS-*Hoxb3* regions chosen for reporter analyses in B,C. Red lines indicate regions with more than 3.0-fold enrichment (ChIP/WCE), *P*<0.0001 (one-sided Wilcoxon test) and a normalized peak intensity of >3.0. (B,C) *EGFP* expression (brackets) in chick forelimb buds driven by BS-*Hoxb3-EGFP* (B) and BS-*Raldh2-EGFP* (C). Embryos were co-electroporated with pCAGGS-*RFP* (B′,C′). Scale bars: 500 μm. (D,E) Occupancy of the BS-*Raldh2* (D) and BS-*Hoxb3* (E) by Cux2 proteins as revealed by ChIP-qPCR analysis of the lateral plate mesoderm from stage 15 chick embryo. Mean±s.d. (*n*=3). (F) Schematic model for the role of *Cux2* in forelimb field specification in chick embryo. Based on our findings, Cux2 directly activates expression of *Raldh2* and *Hoxb3* and possibly other *Hoxb* genes in the forelimb field. We also showed that Cux2 regulates the expression of *Hand2* and *Shh* in the forelimb field. Previous studies showed that retinoic acid (RA) signaling and *Hox* genes are involved in specification of the forelimb field along the anterior-posterior axis (Nishimoto et al., 2015; Waxman et al., 2008; Zhao et al., 2009). It is also shown that axial *Hox9* paralogs establish the posterior forelimb field by triggering posteriorly restricted *Hand2* expression, which directly activates *Shh* at the posterior margin (Xu and Wellik, 2011). Therefore, Cux2 seems to be involved in specification of the forelimb field via multiple pathways, including the regulation of *Hoxb* genes, *Raldh2* and the specification of the polarizing region. See text for more references and details.

To find the active enhancers, the possible target sequences of Cux2 in the lateral plate mesoderm, close to the loci of *HoxB* clusters and *Raldh2*, we dissected the lateral plate mesoderm from 40 chick embryos at HH 15 and performed ChIP-Seq using antibodies against a histone acetylation marker (H3K27ac). Immunoprecipitated DNA fragments were analyzed using massively parallel sequencing, and the resulting 50-bp sequence reads were aligned with the reference chicken genome (*galGal3*). This analysis allowed us to identify sequences enriched with H3K27ac within ±50 kb of the transcriptional start sites (TSSs) of *Hoxb3* and *Raldh2* (Fig. 4A; SRA accession number, SRP075943). We then compared these data with the putative regulatory elements enriched in H3K4me1 previously identified in HH 16 chick embryos (Seki et al., 2017). This allowed us to identify the possible enhancer sequences, 10 kb downstream of *Hoxb3* (BS-*Hoxb3*; Fig. 4A) and within the first intron (23 kb downstream of the TSS) of *Raldh2* (BS-*Raldh*2; Fig. 4A).

We then assessed the enhancer potential of BS-*Raldh2* and BS-*Hoxb3* within the forelimb bud by *in ovo* reporter analysis (Fig. 4B,C). These sequences were cloned upstream of a basal promoter followed by an *EGFP* reporter, and the resulting constructs were introduced into HH 13-14 chick presumptive forelimb fields with a *RFP* vector, and reporter expression was examined at HH 19. Twenty-four hours after electroporation, both BS-*Raldh2-EGFP* and BS-*Hoxb3-EGFP* drove *EGFP* expression in forelimb buds (arrowheads in Fig. 4B,C), suggesting that these sequences have the enhancer activity in forelimb fields.

Finally, we carried out ChIP-qPCR analysis with stage 15 chicken lateral plate mesoderm at the level of somites 21–26 using anti-Cux2 (Fig. 4D,E). ChIP-qPCR analysis showed a distinct degree of enrichment of Cux2 binding to BS-*Raldh2* and BS-*Hoxb3* (Fig. 4D,E). These results suggest that Cux2 directly binds to the BS-*Raldh2* and BS-*Hoxb3* putative enhancers, positively regulating the expression of *Raldh2* and *Hoxb3* in the lateral plate mesoderm.

Taken together, our results indicate that Cux2 refines the forelimb field by controlling transcription of *Hoxb* genes as well as the synthesis of retinoic acid in the lateral plate mesoderm in chicken embryos.

## DISCUSSION

In this study, we have described a novel function for *Cux2* in the refinement of the forelimb field in chicken embryos. Developmental analyses of chick embryos revealed that *Cux2* refines the axial position of the forelimb field. Furthermore, Cux2 directly activates expression of *Hoxb3*, other *Hoxb* genes to a lesser degree, and the retinoic acid synthesis enzyme *Raldh2* in the forelimb field. These results suggest that *Cux2* refines the forelimb position along the rostral-caudal body axis via regulation of *Hoxb* transcription and retinoic acid synthesis (Fig. 4F).

It has been proposed that the nested expression of *Hox* genes in the lateral plate mesoderm is somehow related to the regionalization of the forelimb, interlimb flank and hindlimb fields (Cohn et al., 1997; Nelson, 1994). Anterior *Hox* genes were recently shown to directly activate transcription of *Tbx5* in the forelimb field (Minguillon et al., 2012), whereas posterior *Hox* genes repress *Tbx5* expression to restrict its expression to the forelimb field (Nishimoto et al., 2014). Furthermore, a more recent study showed that in chicken embryos the lateral plate mesoderm compartment, such as the forelimb, interlimb and hindlimb fields, is progressively formed by sequential collinear activation of *Hox* genes during gastrulation (Moreau et al., 2019). Thus, the regulation of *Hox* genes by Cux2 may further restrict *Tbx5* expression to the forelimb field as a late step of positioning of the forelimb. In addition, retinoic acid signaling regulates the expression of *Hox* genes and leads to the regionalization of the lateral plate mesoderm along the rostral-caudal axis (Niederreither et al., 1999; Waxman et al., 2008). Moreover, modulation of retinoic acid signaling during gastrulation affects the axial extent of *Hox* gene expression and leads to changes in the forelimb field in chicken embryos (Moreau et al., 2019). Therefore, retinoic acid, the synthesis of which is activated by Cux2, may also be involved in regulation of *Hox* gene expression in the lateral plate mesoderm during a late step of forelimb positioning. Modulation of *Cux2* expression causes only a slight shift in forelimb position. The data presented here are consistent with previous studies indicating that certain *Hox* gene mutation results in a shift in limb position of only one to a few somite lengths (Favier et al., 1996; Jurberg et al., 2013; McIntyre et al., 2007). Multiple sequences enriched for H3K27ac (SRA accession number, SRP075943) and H3K4me1 (Seki et al., 2017) were identified near the *HoxB* clusters, and thus we cannot exclude the possibility that Cux2 regulates multiple *Hoxb* genes. Consistent with this view, electroporation of pCMV-*hCux2* caused not only the anterior shift of the posterior boundary of the forelimb bud, but also caused posterior extension of the forelimb bud in a single case. Importantly, *Drosophila*, *cut* is involved, directly or indirectly, in the control of expression and/or function of at least two homeotic genes, *proboscipedia* and *Antennapedia* (Johnston et al., 1998).

Therefore, we should also consider the possibility that Cux2 regulates expression of other *Hox* cluster genes and/or posterior *Hox* genes as well.

In this study, we also showed the involvement of *Cux2* in the specification of the posterior forelimb field. Recent analyses of mouse mutants revealed that *Hox9* and *Hox5* genes are involved in the establishment of the posterior and anterior domain**,** respectively, of the forelimb field (Xu et al., 2013; Xu and Wellik, 2011). The *Hox9* paralogous genes trigger posteriorly restricted expression of *Hand2* to establish the posterior forelimb field (Xu and Wellik, 2011). In contrast, *Hox5* genes interact with *Plzf* to cooperatively restrict *Shh* expression to the posterior domain of the forelimb bud (Xu et al., 2013). In this study, misexpression of *Cux2* resulted in the expansion of *Hand2* expression and an anterior shift in *Shh* expression and, in a few cases, reduction of the *Shh* expression domain. This reduction in the region that expresses *Shh* could be caused by ectopically expressed *Hoxb5* genes after *Cux2* misexpression. In addition, we observed two types of transient morphological changes after the misexpression of *Cux2-VP16*: an anterior shift of the forelimb bud and a reduction in the width of the forelimb bud. Because misexpression of *Cux2-VP16* reduced the level of *Shh* expression in a few cases, the width of the shifted forelimb bud may be regulated by the range of signaling from the polarizing region. It is also possible that mosaic and transient expression of *Cux2-VP16* may not be able to continue altering the expression of target genes during the late stages of limb development; instead, the limb bud normalizes. In the early limb field, genetic antagonism between *Hand2* and *Gli3* establishes an anterior-posterior pre-pattern (te Welscher et al., 2002a), and such genetic interplay is also involved in refining the limb position (Rallis et al., 2005). Thus, modification of *Hox* gene expression caused by misexpression of *Cux2* may have caused the anterior expansion of *Hand2* expression and thereby led to the anterior shift in *Shh* expression and of the forelimb bud. In addition, misexpression of *Cux2* showed only mild effects on the expression of *Hand2* and *Shh.* These results are consistent with our view that modification of *Hox* gene expression, which causes only a slight shift of limb position in mutants (Favier et al., 1996; Jurberg et al., 2013; McIntyre et al., 2007), leads to a change in *Hand2* expression. In addition, based on the posteriorly restricted expression pattern of *Cux2* in the limb buds, a potential role for *Cux2* in the establishment of *Shh* expression in the posterior domain of limb buds has been proposed (Tavares et al., 2000).

Retinoic acid signaling is correlated with forelimb initiation and positioning in zebrafish, chick and mouse embryos (Mic et al., 2002; Stratford et al., 1996; Waxman et al., 2008; Zhao et al., 2009). Treatment of chick embryos with the retinoic acid inhibitor disulfiram leads to a disruption of limb formation or a shift in limb position (Stratford et al., 1996). In zebrafish, *raldh2* mutants lack pectoral fin buds (Gibert et al., 2006; Grandel et al., 2002), and embryos treated with retinoic acid inhibitor show a downregulation of *hoxb5b* expression and a failure to induce the formation of pectoral fins (Waxman et al., 2008). Retinoic acid signaling was recently shown to directly activate transcription of *Tbx5* in the forelimb-forming fields (Nishimoto et al., 2015). Thus, we should also consider the possibility that Cux2 contributes to the establishment of the *Tbx5* expression domain in the forelimb field via activation of retinoic acid synthesis. In this study, we showed that Cux2 directly binds to the enhancer of *Raldh2*. However, in certain cases, *Raldh2* expression was altered in non-*Cux2*-transfected regions of the limb buds, suggesting that *Raldh2* expression can also be changed by unknown genes modified after misexpression of *Cux2*. In mouse *Raldh2* mutants, heart-forming fields expand posteriorly and forelimb initiation fails (Mic et al., 2002; Niederreither et al., 1999; Zhao et al., 2009). Thus, retinoic acid signaling seems to have a role in the regionalization of the lateral plate mesoderm into the cardiac and the posterior lateral plate mesoderm. In this study, we introduced *Cux2* constructs into the coelom at the level of the forelimb field, which appears after HH 14. Thus, it is unlikely that the shift in the forelimb position was caused by an alteration of the regionalization into the cardiac and the posterior lateral plate mesoderm, although we cannot exclude the possibility that *Cux2* is involved in this process at much earlier stages.

In this study, we found that *Cux2* expressed in the lateral plate mesoderm refines the position of the forelimb field in chicken embryos. Notably, however, the expression of *Cux2* differs between chicken and mouse embryos, at least in late limb buds (Iulianella et al., 2003). In mice embryos, in contrast to chicken embryos, *Cux2* is expressed in the mesenchyme underlying the apical ectodermal ridge in late limb buds and subsequently in the interdigital region (Iulianella et al., 2003). Although no publications have described the expression or function of *Cux2* in the lateral plate mesoderm prior to the initiation of limb buds, it is possible that the role of Cux2 is not the same in mouse embryos as in chicken embryos with respect to limb positioning.

Importantly, expression of *Cux2* in the hindlimb-forming field is not as strong as in the forelimb field at the pre-limb bud stage. As we mentioned above, the factors involved in specification of the hindlimb field are different from those that specify the forelimb field (Kawakami et al., 2001; Logan and Tabin, 1999; McPherron et al., 1999; Szeto et al., 1999; Xu et al., 2013; Xu and Wellik, 2011). It is, however, possible that factor(s) with similar roles to *Cux2* may be distributed in the hindlimb-forming field.

In conclusion, we revealed a novel function for *Cux2* on refining the forelimb-forming field via regulation of transcription of *Hoxb* gene(s) and retinoic acid synthesis in chicken embryos. As discussed here, retinoic acid and Hox play multiple roles during specification of limb field. Thus Cux2 is likely to be involved in the forelimb specification via multiple pathways, including the specification of the polarizing region and the activation of forelimb initiation gene(s), which future studies should aim to address.

## MATERIALS AND METHODS

No statistical methods were used to predetermine sample size. The experiments were not randomized and the investigators were not blinded to allocation during experiments and outcome assessment. The age of each specimen is noted in the figure legends. The sex of the embryos is unknown.

### Ethics statement

All experiments were performed in accordance with guidelines for animal experiments of Tokyo Institute of Technology.

### Wholemount *in situ* hybridization

Fertilized White Leghorn chicken (*Gallus gallus*) eggs were incubated at 38°C in a humidified incubator and staged according to Hamburger-Hamilton's staging (Hamburger and Hamilton, 1951). *Gallus gallus Cux2* [730 base pairs (bp)], *Hoxb3* (734 bp), *Hoxb5* (717 bp) and *Raldh2* (1500 bp) were amplified from cDNA pools prepared from HH 20 chick embryos using the following primers, which hybridized to the indicated published sequences: *Cux2* (GenBank accession number, XM415167.4), 5′-CCAGGGCAGTGTGAGTGACATGC-3′ and 5′-CCCTTGGCTTCTTGATCTGCAGG-3′; *Hoxb3* (GenBank accession number, NM_204743), 5′-GTCAAAAGGGATGGGCTCTT-3′ and 5′-CTTGGAAACTGTGCCAAACAG-3′; *Hoxb5* (GenBank accession number, NM_001025355), 5′-AGGACAGCGTACACTCGCTAC-3′ and 5′-ACTGCGACTGTAGTGCAGGAAC-3′; *Raldh2* (GenBank accession number, NM_204995), 5′-ATGGCATCTCTGCATCTGCTG-3′ and 5′-TTAGGAATTCTTCTGAGGGATC-3′. Wholemount *in situ* hybridization was carried out essentially as described (Izpisua-Belmonte et al., 1993). Probe templates for *Fgf8*, *Shh* and *Hoxb9* were described previously (Burke et al., 1995; Crossley et al., 1996; Riddle et al., 1993). Some wholemount *in situ* samples were embedded in 2% agarose in phosphate-buffered saline (PBS), and sections were cut at 100 μm thickness with a MicroSlicer ZERO1N (Dosaka EM).

### siRNA preparation

siRNA specific for chicken *Cux2* were obtained from Sigma-Aldrich (siRNA Duplex), and a control siRNA were obtained from Invitrogen (Stealth^TM^ RNAi). The targeted sequence was 5′-CCUACCUGAAGCGUCGGUAUGGGCU-3′ of chick *Cux2*, which corresponds to nucleotides 3215–3239, the homeodomain-encoding region (GenBank accession number, XM415167.4). For negative control, siRNA Negative Control Hi GC Duplex #2 (Invitrogen) was used.

### Plasmid construction

For pCMV-*Cux2*-*EGFP,* the cDNA sequence encoding amino acid residues 870–1113 of chick Cux2 (XP_415167.4) was amplified with the polymerase chain reaction (PCR) using the primers 5′-CCGCTCGAGCCCACCATGGGCAGTGTGAGTGACATGCTG-3′ and 5′-CGGGATCCGGCTTCTTGATCTGCAGGAGG-3′, inserted into the pGEM-T easy vector (Promega) and then cloned into the *Xho*I and *Bam*I sites of pAc*GFP*-N1 (Clontech). For pCMV-*hCux2,* the cDNA for the entire human *Cux2* coding region was obtained from pF1KSDA0293 (Kazusa DNA Research Institute, human clone KIAA0293) and cloned into the *Sgf*I and *Pme*I sites of the pF4A-pCMV Flexi vector (Promega). For pCAGGS-*hCux2*-*VP16*, RCASBP(A)-*Tbx5*-*VP16* (CT#630), a gift from Cliff Tabin (Addgene, Plasmid #13969) (Rallis et al., 2003), was digested with *Cla*I, cloned into the *Cla*I site of the SLAX12NCO vector (Morgan and Fekete, 1996) and then digested with *Eco*RI and *Hin*dIII; the resulting 2×*VP16* fragment was cloned into the pBKKS vector (pBKKS-2×*VP16*). The cDNA sequence encoding amino acid residues 889–1228 of human Cux2 was cloned into the *Spe*I and *Eco*RI sites of pBKKS- duplex *VP16* (pBKKS-Δ*hCux2*-2×*VP16*). The cDNA fragment of Δ*hCux2*-2×*VP16* was amplified using primers including the *Xho*I sequence (5′-TTTGGCAAAGAATTCCTCGAGCCCACCATGGAGCTGTACAT-3′ and 5′-CTGAGGAGTGAATTCCTCGAGGTCGACGGTATCGATAAGC-3′) and inserted into the *Xho*I site of pCAGGS vector (Niwa et al., 1991) via the In-Fusion reaction (Clontech). For enhancer analysis, Binding Sequence (BS)-*Raldh2* and BS-*Hoxb3* were isolated from the chick genome by PCR. The following forelimb forward and reverse primers were used: BS-*Raldh2*, 5′-GCACATACATGACACACCGT-3′ and 5′-CATAGATATTCCTACCACTAAG-3′; BS-*Hoxb3*, 5′-CATAGATCTGCAACACTTCA-3′ and 5′-CCGCTTCGATTCCTTTCC-3′. These sequences were subcloned in front of a chicken β-actin basal promoter (Matsuo et al., 1991), that is followed by a *GFP* reporter (Ogino et al., 2008).

### Cell culture and transfection

COS7 cells provided by M. Komada (Tokyo Institute of Technology, Yokohama, Japan) (Reincke et al., 2015), were maintained in DMEM (Nacalai tesque) supplemented with 10% fetal bovine serum (Gibco Invitrogen), 100 U/ml penicillin and 100 μg/ml streptomycin (Wako). Co-transfection of target plasmid pCMV-*Cux2*-*EGFP* (encoding enhanced green fluorescent protein, EGFP) and siRNAs was carried out with Polyethylenimine ‘MAX’ (Polysciences Inc.) as per the manufacturer's protocol. To assess the efficiency of the siRNAs on pCMV-*Cux2*-*EGFP* expression in COS7 cells, the number of EGFP-positive cells was counted at 24 h after transfection. Student's *t*-test was performed to assess differences in the number of EGFP-positive cells between cells transfected with *Cux2* and control siRNAs (*P*<0.00001).

### *In ovo* DNA electroporation

Plasmid or siRNA solution was colored with 1–5% Fast Green and co-electroporated with pCAGGS-*EGFP* (a gift from Dr Miyazaki and Dr Ogura) (Niwa et al., 1991) or pCAGGS-*RFP* (Das et al., 2006) (a gift from Dr Tickle) into the presumptive limb mesenchyme as described (Onimaru et al., 2015; Suzuki and Ogura, 2008). Briefly, the solution was injected into the lateral plate mesoderm of HH 13–14 chick embryos, and electroporated with two pulses of 8–12V, 85 ms by CUY21 EDIT (BEX Co., Ltd), or five pulses of 6V, 20 ms after a poration pulse of 25V, 0.05 ms by CUY21 EDIT II (BEX Co., Ltd.). The concentrations of the prepared plasmid solutions or siRNAs were as follows: pCAGGS-*EGFP*, 1–5 mg/ml; pCMV-*hCux2*, 5 mg/ml; pCAGGS-*hCux2-VP16*, 5 mg/ml; control siRNA, 5 μM and *Cux2* siRNA, 250 μM. For *in ovo* enhancer analysis, 5–10 mg/ml of BS-*Hoxb3* or BS-*Raldh2* was electroporated into the lateral plate mesoderm of HH 13–14 chick embryos.

### Analysis of the position of the forelimb bud

Measurements were made using ImageJ (https://imagej.net/Downloads). The length of both the AER and the region between seven somites including the limb field were measured in embryos stained with *fgf8* RNA probes. Half of the distance between the seven somites was defined as ‘zero’ and used as a reference to measure the relative position of the limbs.

### Cartilage staining

The morphology of the limbs that were subjected to experimental manipulation was studied after cartilage staining with Alcian Blue (Tanaka et al., 2000).

### Chromatin immunoprecipitation sequencing (ChIP-seq) and quantitative real-time PCR (ChIP-qPCR)

ChIP was performed as described (Nakato et al., 2013; Suda et al., 2014; Visel et al., 2009). Briefly, the lateral plate mesoderm (at the level of somites 21–26) from 40 HH-15 chick embryos was dissected, fixed in 1% formaldehyde for 10 min at room temperature, washed with PBS and stored at −80°C. ChIP was performed from these tissue samples using antibodies against H3K27ac (ab4729, Abcam) or Cux2 antibodies (Eurofins Genomics K. K., Tokyo, Japan). To generate polyclonal antibodies against chicken Cux2, two synthetic peptides, N-SAGSDSESPGARSEC-C and N-C+RLSTSVQRRHEKMA-C, derived from estimated amino acids 1080–1094 and 1346–1359, respectively (GenBank accession number, XP_415167.4), were synthesized, conjugated to keyhole limpet hemocyanin and used to immunize rabbits; the resulting antisera were purified by affinity chromatography (Eurofins Genomics K.K., Tokyo, Japan).

DNA samples from the whole-cell extract (WCE) and ChIP fractions were further sheared with an ultra sonicator (Branson Sonifire 250D), ligated to sequencing adapters and amplified according to the manufacturer's instructions (Applied Biosystems SOLID 5500). Gel-purified amplified DNA (100–150 bp) was sequenced on an Applied Biosystems SOLID 5500 platform to generate 50-bp reads. Sequence reads were aligned with the *G. gallus* reference genome (*galGal*3) with Bowtie version 1.1.2 (Langmead et al., 2009), allowing three mismatches per read and outputting only uniquely mapped reads (-n3 -m1 option). The mapping statistics are summarized in Table S1. More than 10 million reads were mapped for each sample. For peak calling and data visualization, we used DROMPA (Nakato et al., 2013) with a parameter set that identified the regions that satisfied the following criteria: >3.0-fold enrichment (ChIP/WCE), *P*<1×10^–4^ (one-sided Wilcoxon test) and a normalized peak intensity of >3.0. ChIP-Seq from this study are available from the Sequence Read Archive (SRA) database (http://www.ncbi.nlm.nih.gov/sra) under the accession number SRP075943.

For ChIP-qPCR, DNA samples from the WCE and ChIP fractions were sheared with the ultra sonicator, incubated with Cux2 antibodies (Eurofins Genomics K. K., Tokyo, Japan) and purified. Putative binding site sequences were amplified with the following primers (BS-*Raldh2*, 5′-GTAGCATGATTTACATGGAAGC-3′ and 5′-GTGACCGTGGTAAAGGCTAAC-3′; BS-*Hoxb3*, 5′-TCTCAGGAATCAGAATGAGCC-3′ and 5′-GCGCTTCCCTCGGTTTTATA-3′). The mean±standard deviation (s.d.) was calculated, and a statistical analysis was performed using Student's *t*-test.

## Supplementary Material

Supplementary information
