# Supplementary material for: Cux2 refines the forelimb field by controlling expression of Raldh2 and Hox genes
Source: Biol Open. 2019 Jan 16;8(2):bio040584. doi: 10.1242/bio.040584 (PMC6398465; doi:10.1242/bio.040584)
Supplement: Supplementary information [file biolopen-8-040584-s1.pdf]

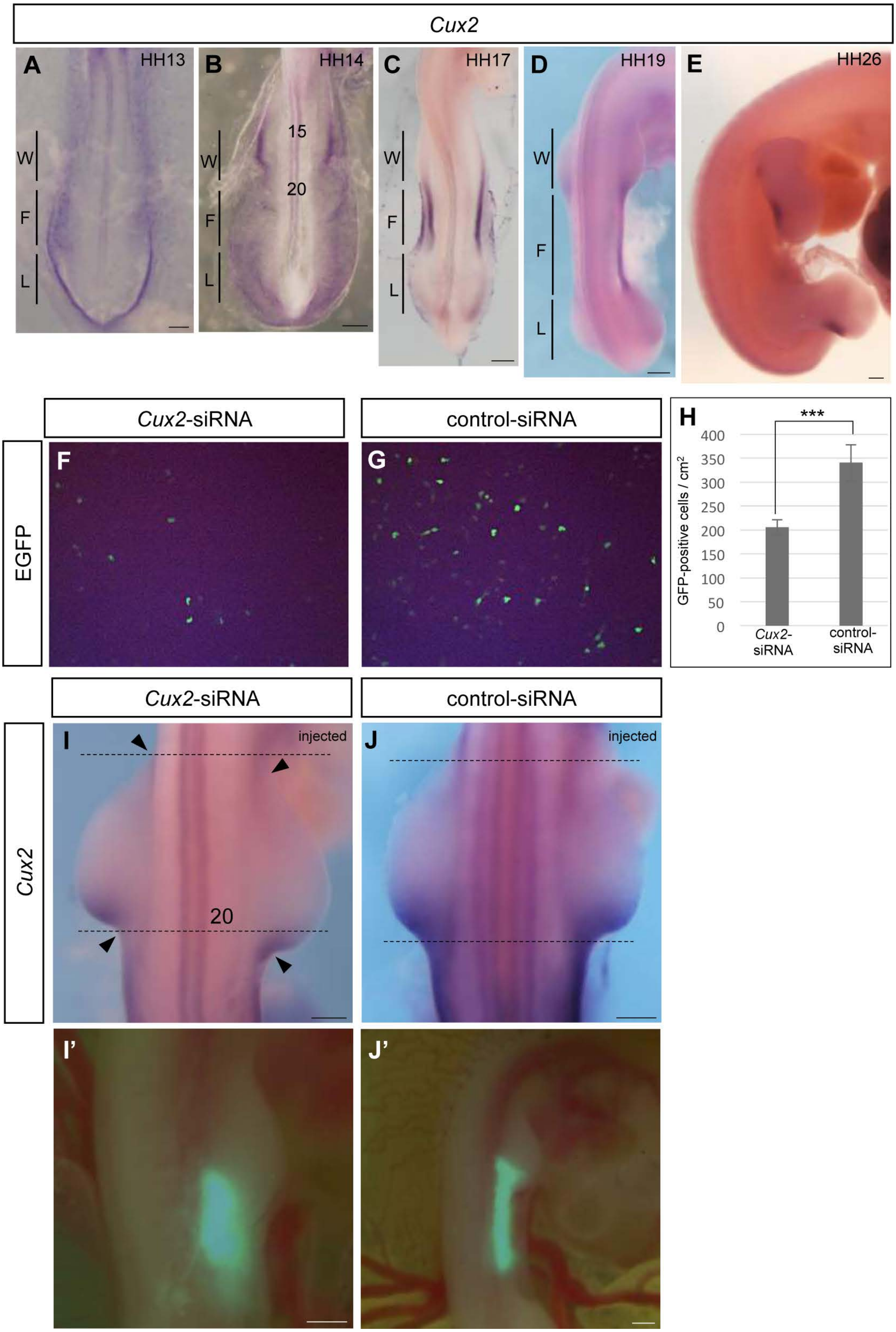

Biology Open • Supplementary information

Fig. S1. Expression of Cux2 and depletion of Cux2 by Cux2-siRNA. (A-E) Expression of *Cux2* in developing chick embryos at HH 13 (A), 14 (B), 17 (C), 19 (D) and 26 (E). (F, G) pCMV-*Cux2-EGFP* and either *Cux2*-siRNA (F) or control-siRNA (G) were co-transfected into COS7 cells. (H) EGFP-positive cells were counted at 24 hours after transfection. (I, J) Expression of *Cux2* in chick forelimb buds after the electroporation of either *Cux2*-siRNA (I) or control-siRNA (J) into the presumptive right forelimb buds. Black dotted lines indicate the width of the left (control side) forelimb bud. Notably, Cux2 expression was downregulated in the forelimb bud at the somite 20 level at the *Cux2*-siRNA-injected site (I). (I', J') pCAGGS-*EGFP* was used to assay efficiency of electroporation. White dotted lines indicate the outlines of the right forelimb bud. Numbers in (B) and (I) indicate somite levels. Scale bars, 500 µm.

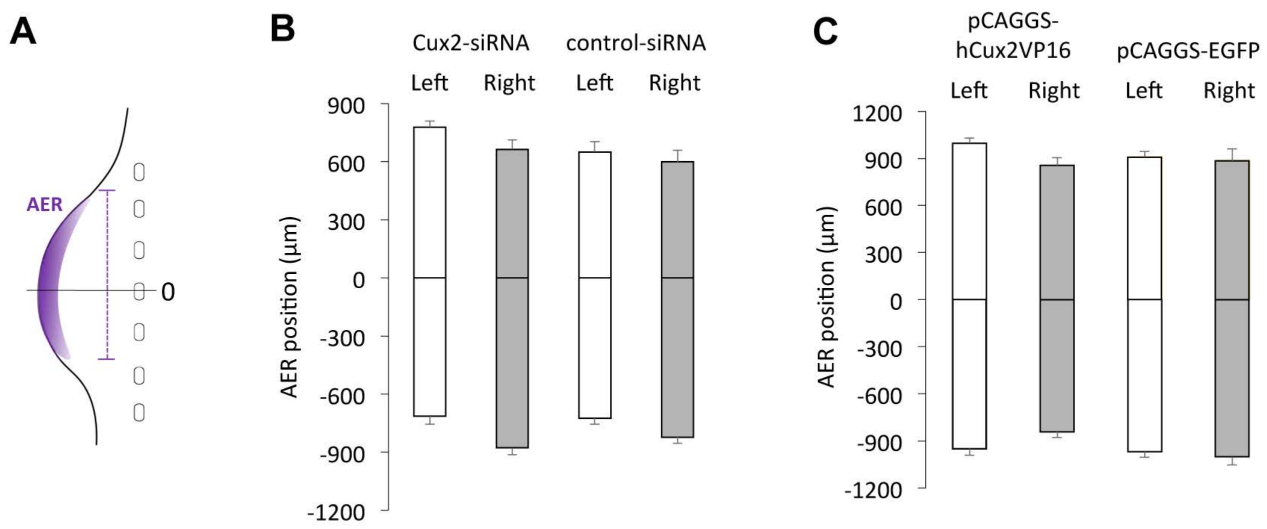

Fig. S2. Analysis of the position of the forelimb buds electroporated with Cux2-siRNA, control-siRNA, or pCAGGS-hCux2-VP16, together with pCAGGS-EGFP, or pCAGGS-EGFP alone. (A) The position of the AER was measured in embryos stained with *fgf8* probes. The length of the region between seven somites including the forelimb was measured, and its center ("zero") was used as a reference for the AER position along the anterior-posterior axis. (B) Forelimb buds electroporated with *Cux2*-siRNA are shifted posteriorly. (C) Forelimb buds electroporated with pCAGGS-*hCux2-VP16* are reduced and an anterior shift of the posterior limb boundary is observed. Mean ± s.e.m.

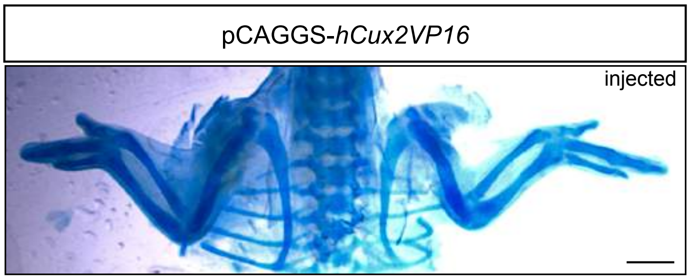

Fig. S3. Cartilage patterns of forelimbs electroporated with pCAGGS-hCux2-VP16. Cartilage patterns of forelimbs electroporated with pCAGGS-*hCux2*-*VP16*. Scale bars, 2 mm.

Biology Open • Supplementary information

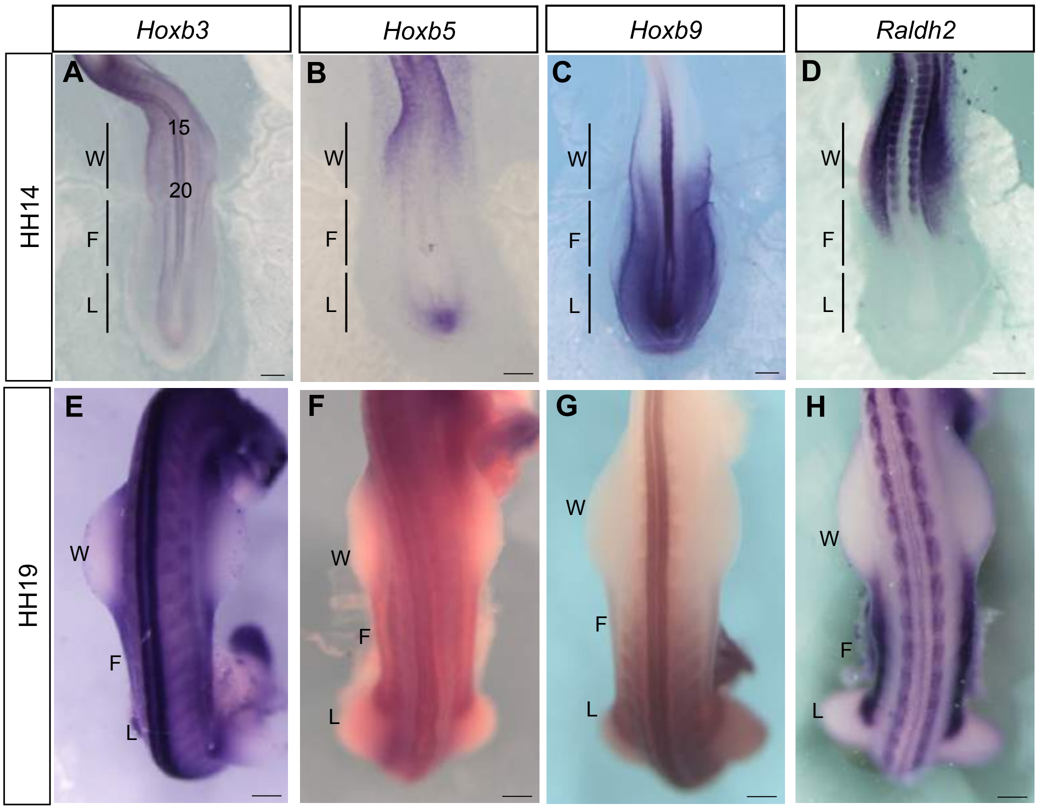

Fig. S4. Expression of Hoxb3, b5, b9 and Raldh2. (A-H) Expression of *Hoxb3* (A, E), *b5* (B, F), *b9* (C, G) and *Raldh2* (D, H) in developing chick embryos at HH 14 (A-D) and 19 (E-H). Scale bars, 500 µm.

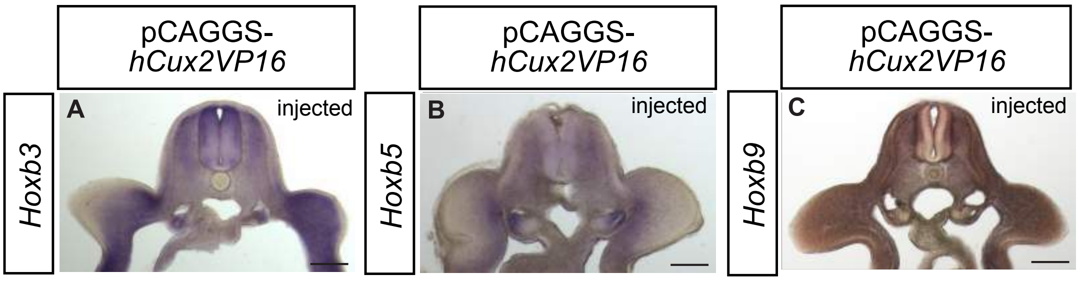

Fig. S5. Cux2 regulates the expression of Hoxb genes. (A-C) Transverse sections of the forelimb buds hybridized with *Hoxb3* (A)*, Hoxb5* (B), and *Hoxb9* (C), respectively, after electroporation with pCAGGS-*hCux2-VP16*. Scale bars, 200 μm.
